# Pericarpium Trichosanthis Injection Protects Isoproterenol-Induced Acute Myocardial Ischemia via Suppressing Inflammatory Damage and Apoptosis Pathways

**DOI:** 10.3390/biom15050618

**Published:** 2025-04-24

**Authors:** Zizheng Wu, Xing Chen, Jiahao Ye, Xiaoyi Wang, Zhixi Hu

**Affiliations:** College of Chinese Medicine, Hunan University of Chinese Medicine, No. 300, Bachelor Road, Hanpu Science and Education Park, Yuelu District, Changsha 410208, China; wuzizheng@stu.hnucm.edu.cn (Z.W.); 20242097@stu.hnucm.edu.cn (X.C.); 20193211@stu.hnucm.edu.cn (J.Y.); wangxy@stu.hnucm.edu.cn (X.W.)

**Keywords:** pericarpium trichosanthis injection (PTI), acute myocardial ischemia, network pharmacology, molecular docking, apoptosis, isoproterenol, rats

## Abstract

This research proposes to systematically investigate the cardioprotective mechanisms of *Pericarpium Trichosanthis* injection (PTI) against acute myocardial ischemia through an integrated approach combining ultra-performance liquid chromatography quadrupole time-of-flight mass spectrometry (UPLC-Q-TOF/MS) constituent profiling, UNIFI database-assisted component identification, network pharmacology-guided target prediction, molecular docking verification, and in vivo experimental validation. The multimodal methodology is designed to comprehensively uncover the therapeutic benefits and molecular pathways underlying this traditional Chinese medicine formulation. Methods: UPLC-Q-TOF/MS and the UNIFI database were used in conjunction with a literature review to screen and validate the absorbed components of PTI. Using network pharmacology, we constructed protein-protein interaction (PPI) networks for pinpointing prospective therapeutic targets. In addition, Gene Ontology (GO) and Kyoto Encyclopedia of Genes and Genomes (KEGG) analyses were performed to identify potential signaling pathways. In vivo experiments were conducted to investigate the mechanisms by which PTI ameliorated isoproterenol-induced myocardial injury in rats. All animal experiments have adhered to ARRIVE guidelines. Results: UPLC-Q-TOF/MS revealed 11 core active components in PTI. Network pharmacology prioritization identified pseudoaspidin, ciryneol C, cynanoside M, daurinol, and n-butyl-β-D-fructopyranoside as central bioactive constituents within the compound-target interaction network. Topological analysis of the protein interactome highlighted AKT1, EGFR, MMP9, SRC, PTGS2, STAT3, BCL2, CASP3, and MAPK3 as the most interconnected nodes with the highest betweenness centrality. Pathway enrichment analysis established the PI3K/Akt signaling cascade as the principal mechanistic route for PTI’s cardioprotective effects. Molecular docking simulations demonstrated high-affinity interactions between characteristic components (e.g., cynanoside M, darutigenol) and pivotal targets including PTGS2, MAPK3, CASP3, and BCL2. In vivo investigations showed PTI treatment markedly attenuated myocardial tissue degeneration and collagen deposition (*p* < 0.05), normalized electrocardiographic ST-segment deviations, and suppressed pro-inflammatory cytokine production (IL-6, TNF-α). The formulation concurrently reduced circulating levels of cardiac injury indicators (LDH, cTnI) and oxidative stress parameters (ROS, MDA), Regarding apoptosis regulation, PTI reduced Bax, caspase-3, and caspase-9, while elevating Bcl-2 (*p* < 0.05), effectively inhibiting myocardial cell apoptosis with all therapeutic outcomes reaching statistical significance. These findings highlight PTI’s protective effects against myocardial injury through multi-target modulation of inflammation, oxidation, and apoptosis. Conclusions: PTI exerts its therapeutic effects in treating acute myocardial ischemia by regulating and suppressing inflammatory responses, and inhibiting cardiomyocyte apoptosis.

## 1. Introduction

Myocardial ischemia, a condition marked by diminished heart blood flow from coronary artery narrowing, can lead to angina pectoris, myocardial infarction, heart failure, and sudden cardiac death [1]. In the current era, its incidence has significantly increased in China, posing a substantial threat to public health [2]. Research has demonstrated that oxidative stress and inflammatory responses induced by excessive reactive oxygen species (ROS) production in ischemic myocardium exacerbate myocardial injury, representing critical pathophysiological mechanisms in myocardial ischemia [3]. Growing evidence also highlights the involvement of apoptosis in the progression of this condition [4]. Significantly, pro-inflammatory cytokines (e.g., TNF-α, IL-6) released during inflammatory cascades activate the Caspase-dependent apoptotic pathway in cardiomyocytes. Concurrently, impaired clearance of apoptotic cells amplifies inflammatory responses through secondary necrosis, establishing a self-reinforcing “inflammation-apoptosis” circuit that accelerates the progression of myocardial ischemia toward irreversible tissue damage [5].

Current primary therapies for myocardial ischemia include β-blockers (e.g., metoprolol, propranolol) [6], which are associated with risks of respiratory complications [7]. Therefore, identifying effective treatments with minimal adverse effects is of paramount importance. Traditional Chinese medicine (TCM), recognized for its multi-target pharmacological properties, low toxicity, and favorable safety profile, has garnered increasing attention [8]. Extensive studies on bioactive components and their mechanisms have facilitated the clinical application of TCM in myocardial ischemia [9].

*Pericarpium trichosanthis* injection (PTI), sourced from *Trichosanthes kirilowii* Maxim’s mature dried fruit peel, serves as an injectable. It has been widely used in the clinical management of cardiovascular diseases, including coronary heart disease, unstable angina, and coronary artery disease [10]. Recent studies indicate that PT exerts cardioprotective effects by suppressing inflammation, inhibiting apoptosis, and ameliorating myocardial fibrosis [11,12]. However, the precise mechanisms underlying PTI’s therapeutic efficacy in myocardial ischemia remain unclear.

Therefore, Leveraging network pharmacology—a discipline integrating systems biology, network analysis, and multi-target drug discovery—this study aimed to elucidate PTI’s therapeutic mechanisms [13]. Initially, ultra-high-performance liquid chromatography-quadrupole time-of-flight mass spectrometry (UPLC-Q-TOF/MS) coupled with the UNIFI database was used to identify and confirm blood-absorbable bioactive components of PTI. Subsequently, a “drug-active ingredient-target-pathway” network model was constructed to systematically predict PTI’s potential targets and signaling pathways in acute myocardial ischemia using network pharmacology and molecular docking. Finally, In vivo studies were conducted in an isoproterenol-induced animal model to confirm the pharmacodynamic basis and theoretical rationale of PTI for clinical application. This integrative approach not only clarifies PTI’s unique multi-component, multi-target mechanisms but also provides a novel framework for translating traditional Chinese medicine into evidence-based therapy.

## 2. Materials and Methods

### 2.1. Animals

A grand total of 60 SPF-grade male Sprague-Dawley (SD) rats, aged between 6 and 8 weeks and weighing around 200 ± 20 g, were sourced from Hunan Slake Jingda Experimental Animal Co., Ltd., Changsha, China (catered by license SCXK2019-0004). These rodents were kept at the Animal Experimental Center of Hunan University of Traditional Chinese Medicine, where they were subjected to a controlled environment: a temperature of 22 ± 2 °C, humidity levels ranging from 50 to 70%, and a 12-h light/dark schedule. They had unlimited access to both food and water. The Institutional Animal Ethics Committee of the Hunan University of Chinese Medicine gave the green light to approve all experimental protocols. s. (approval no. HNUCM21-2403-54, date: 20 March 2024) and conducted in compliance with ARRIVE guidelines.

### 2.2. Reagents

The following reagents were used in this study: rat superoxide dismutase (SOD) detection ELISA kit (catalog no: AF3262-A, suppliers: AiFang biological, Changsha, China), rat reactive oxygen species (ROS) ELISA kit (catalog no: AF3686-A, suppliers: AiFang biological, Changsha, China), rat tumor necrosis factor-α (TNF-α) ELISA kit (catalog no: AF3056-A, suppliers: AiFang biological, Changsha, China) rat lactate dehydrogenase (LDH) ELISA kit (catalog no:AF3480-A, suppliers: AiFang biological, Changsha, China), rat malodialdehyde (MDA) ELISA kit (catalog no: AF8503-A, suppliers: AiFang biological, Changsha, China), rat cardiac troponin I (cTnI) ELISA kit (catalog no: AF3686-A, suppliers: AiFang biological, Changsha, China), hypersensitive C reactive protein (hs-CRP) ELISA kit (catalog no: AF3246-A, suppliers: AiFang biological, Changsha, China), rat Interleukin-1β (IL-1β) ELISA kit (catalog no:AF2923-A, suppliers: AiFang biological, Changsha, China), rat Interleukin-6 (IL-6) ELISA kit (catalog no: AF3066-A, suppliers: AiFang biological, Changsha, China), Bax Polyclonal Antibody (catalog no:AF01639, suppliers: AiFang biological, Changsha, China), BCL-2 mouse monoclonal antibody (2F1) (catalog no:AF11892, suppliers: AiFang biological, Changsha, China), Caspase-3 polyclonal antibody (catalog no: AF06646, suppliers: AiFang biological, Changsha, China), GAPDH Monoclonal Antibody (catalog no: YM3029, suppliers: Immunoway, San Diego, CA, USA) cleaved-Caspase-9p35 (D315) polyclonal antibody (catalog no: AF00014, suppliers: AiFang biological Changsha, China), TUNEL cell apoptosis detection kit (catalog no: AFIHC032, suppliers: AiFang biological Changsha, China), methanol (catalog no: MS1922-801, suppliers: TEDIA, Fairfield, OH, USA), and acetonitrile (catalog no:AS1122-801, suppliers: TEDIA, Fairfield, OH, USA).

### 2.3. Instruments

The following tools were used in this study: Waters ACQUITY UPLCI-Class/XEVO G2-XS Qtof-MS (Waters, Milford, MA, USA.; UNIFI v1.8 Portal Scientific information system(Waters, Milford, MA, USA); Masslynx V4.2 mass spectrum workstation (Waters, Milford, MA, USA); Microsoft Office Excel 2016; Water Bath-Slide Drier (Jinhua Kdy Instrument and Equipment Co., Ltd., model number: KD-P, Jinhua, China); staining machine: (DIAPATH, model number: Giotto, Milan, Italy); oven (Tianjin LeiRui Instrument Co., Ltd., Tianjin, China, Model: GFL-230); slides (Servicebio, model number: G6004, Wuhan, China).

### 2.4. Drugs

PTI (Chinese medicine approval Z20027540, Lot: 23081512, suppliers: Shanghai Shangyao First Biochemical Pharmaceutical Co., Ltd., Shanghai, China), propranolol hydrochloride tablets (Pro) (catalog no: E231235, suppliers: Changzhou Yabang Pharmaceutical Co., Ltd., Changzhou, China), and isoproterenol HCl (catalog no: F2215417 suppliers: Aladdin, Shanghai, China) were used in this study.

### 2.5. UPLC-Q-TOF/MS Analysis

#### 2.5.1. Preparation of Drug and Drug-Containing Serum

##### Preparation of the Test Solution

20 μL of PTI was mixed with 980 μL of ultrapure water (UPW), and the mixture was filtered through a 0.22-μm microporous membrane.

##### Serum Injection

Following a 7-day acclimatization period, interspecies dose conversion was performed via body surface area normalization (animal-to-human equivalent dose calculation). The experimental protocol comprised: PTI group: 4.8 mL/kg/day given once daily for 7 days. Control group: Equivalent volume of vehicle control (0.9% NaCl) delivered under an identical regimen. Twenty-four hours post-final fasting, blood samples were obtained via abdominal aortic puncture under isoflurane inhalation anesthesia (induction: 3–4%, maintenance: 2–2.5%), followed by euthanasia via cervical dislocation in compliance with ethical guidelines. Serum metabolites were extracted through standardized protocols: 500 μL serum was mixed with 1500 μL pre-chilled (−20 °C) methanol:acetonitrile (1:1, *v*/*v*), vortex-mixed (3 min, 25 °C), and centrifuged (13,000× *g*, 10 min, 4 °C). The supernatant (1500 μL) underwent nitrogen blowdown to complete dryness, reconstituted in 70% methanol aqueous solution, vortexed (3 min) and re-centrifuged (13,000× *g*, 15 min, 4 °C). The resulting supernatant was transferred to LC-MS vials for chromatographic separation using a Waters ACQUITY UPLC BEH C18 column (2.1 × 100 mm, 1.7 μm) coupled with a UPLC-QTOF/MS system.

#### 2.5.2. Analysis of PTI Blood Components

The chromatographic analysis was performed using an ACQUITY UPLC BEH C18 reversed-phase column (50 mm length × 2.1 mm internal diameter) packed with 1.7 μm stationary phase particles. The chromatographic eluent system comprised two components: Mobile phase A (0.1% formic acid aqueous solution) and phase B (acetonitrile). A multi-step gradient protocol was implemented: Initial 0–1 min at 2% B; linear ramp to 60% B over 1–6 min; stepped increase to 90% B from 6–13 min; accelerated transition to 100% B during 13–14 min; isocratic hold at 100% B for 14–15 min; rapid re-equilibration to 2% B between 15–17 min; followed by 2-min column stabilization. Operational parameters included 0.35 mL/min flow rate and 1 μL injection volume.

The mass spectrometry system employed an electrospray ionization (ESI) interface configured for dual-polarity scanning (ESI+/ESI−). Key parameters included a full-scan mass range of *m*/*z* 100–1500, capillary voltages set at 3.0 kV (ESI+) and 2.5 kV (ESI−), a nozzle potential of 40 V, and a drying gas flow rate of 800 L/h. The ionization module was thermally regulated at 120 °C with argon collision gas, and collision-induced dissociation energies were optimized between 20–50 eV.

#### 2.5.3. Screening of Effective Blood Entry Components

PTI, blank serum, and serum samples were analyzed using UNIFI v1.8 software. The database was developed based on a review of PubChem, Scifinder, Chemicalbook, and UNIFI v1.8 Chinese Medicine databases and a literature review. The processing of LC-MS raw data, extraction of feature peaks, and their identification were conducted using Masslynx. Raw data were imported into UNIFI v1.8 software to identify feature peaks, fit isotope distribution, and match the elemental composition. An exact quality number screening template was selected based on the MSE data. The method parameters were set as follows: the threshold for 2D peak detection was 500, the 3D peak scan threshold for low energy was 300, the threshold for the high-energy scan was 40, and the corrected masses were 556.2766 and 554.2620 for the positive and anionic modes, respectively. For components with a retention time in the database, the target filter settings were as follows: the retention time matching threshold was set to ±0.1 min, the parent ion mass deviation threshold was set to ±10 ppm, the predicted fragment ion matching tolerance generated from the structure was set to 10 mDa, and the maximum number of fragment ions was set to 10. The optimized database was imported into this method. The settings for the current data processing methods were saved for data analysis.

UNIFI v1.8 software automatically matches the molecular ion peaks identified by the low-energy channels with the established database and then automatically lists the best matching materials with the fragment ion peaks matched by the high-energy channels. Physical and chemical characteristics, including chromatographic retention time, exact molecular mass, distinctive fragment ions, and polar differences within the same lineage, were chosen for manual confirmation and enhanced accuracy. Simultaneously, reliable identification results of the characteristic peaks were annotated by comparing the compound characteristics with the relevant literature.

### 2.6. Network Pharmacology Analysis

#### 2.6.1. Collection of Active Ingredients and Targets of PTI

The pertinent simplified molecular-input line-entry system (SMILES) number was obtained from the PubChem database. Subsequently, relevant targets were predicted by means of the Swiss Target Prediction database.

#### 2.6.2. Prediction of Acute Myocardial Ischemia-Related Targets

To investigate acute myocardial ischemia, the DisGeNET database at https://www.disgenet.org (accessed on 24 May 2024) and the GeneCards database at http://www.genecards.org (accessed on 24 May 2024) were accessed. We set the GeneCards relevance score at 10 and the DisGeNET score at 0.1 as our benchmarks.

#### 2.6.3. Computational Construction of Protein-Protein Interaction (PPI) Networks

Protein interaction analysis was performed using the STRING database (https://www.string-db.org/ (accessed on 24 May 2024)) with targets identified from “PTI-acute myocardial ischemia” research. The platform was configured for Homo sapiens proteome analysis with multi-protein query mode. The PPI was developed using a medium confidence score cutoff of 0.4 to identify potential therapeutic targets for acute myocardial ischemia. Resultant data were exported as tsv format files, with subsequent topological analysis of hub targets conducted via Cytoscape 3.7.2. For network pharmacology parameterization, the interaction score cutoff was systematically evaluated: initial high-stringency settings (0.7–0.9 confidence levels) excluded critical low-scoring interactions, as evidenced by reduced node connectivity and diminished degree centrality. Therefore, an optimized interaction score threshold of 0.4 was implemented to enhance network inclusivity while maintaining biological relevance.

#### 2.6.4. Computational Framework for Multilayer Drug-Active Ingredient-Target Interaction Mapping

The aforementioned PTI-derived bioactive constituents, therapeutic targets, and associated pathway-disease correlations were subjected to network visualization using Cytoscape 3.7.2. An integrated network model delineating the “PTI components—bioactive molecules—shared targets” axis in acute myocardial ischemia was established, with systematic evaluation of topological attributes including node degree centrality and betweenness centrality for network pharmacology characterization.

#### 2.6.5. Functional Enrichment Analysis of Gene Ontology (Gene Ontology, GO) and Pathway Enrichment Analysis of Kyoto Encyclopedia of Genomes (Kyoto Encyclopedia of Genes and Genomes, KEGG)

The therapeutic targets associated with PTI intervention in acute myocardial ischemia were processed through the Bioconductor platform under RX64 4.0.4 computational environment. Functional genomics analyses were conducted encompassing GO annotation and KEGG pathway enrichment profiling, with a specific focus on hub targets. Multimodal visualization outputs including histogram-based prioritization charts and multidimensional enrichment bubble plots were generated to delineate functional hierarchies.

#### 2.6.6. Molecular Docking Validation

The primary bioactive constituents identified in the “PTI-active component-common target” network were subjected to ligand-receptor binding simulations. Target protein 3D models were sourced from the Protein Data Bank (PDB, https://www.rcsb.org/ (accessed on 24 May 2024)), followed by the removal of non-essential ligands and solvent molecules via PyMol. Small molecule SDF ligand files (PubChem: https://pubchem.ncbi.nlm.nih.gov/ (accessed on 24 May 2024)) were converted to Mol2 format using OpenBabel 3.1.1. Proteins underwent structural optimization in AutoDockTools-1.5.6, including hydrogen atom addition and partial charge calculation, before being exported as PDBQT files. Molecular docking was executed in AutoDock Vina, with subsequent visualization and interaction analysis performed in Discovery Studio-4.5.

### 2.7. In Vivo Studies

#### 2.7.1. Groups, Model, and Doses

Following a 7-day habituation phase, sixty pathogen-free Sprague-Dawley rats were stratified into six experimental cohorts using a random number table generated by Microsoft Excel, with 10 rats in each group (cage positions were scrambled every 3 days to reduce environmental bias), as follows: the control group (n = 10) received oral gavage of normal saline (10 mL/kg) daily for 7 days, with equal-volume saline injected subcutaneously at two dorsal points during the modeling phase; the model group (n = 10) was treated identically to the control group except for subcutaneous injection of ISO (isoproterenol hydrochloride, 85 mg/kg dissolved in saline) on days 6 and 7 (24-h interval); the propranolol group (n = 10) received oral gavage of propranolol solution (10 mg/kg, prepared by dissolving propranolol hydrochloride tablets in ultrapure water) daily for 7 days with the same modeling procedures as the model group; the low-, medium-, and high-dose PTI groups (PTI-L/M/H, n = 10/group) received oral gavage of PTI aqueous extract at 1.2, 2.4, and 4.8 mL/kg daily for 7 days, respectively, with modeling procedures identical to the model group. The PTI dosage regimen was calculated through interspecies equivalent dose ratio conversion based on body surface area (BSA) normalization between human subjects and experimental animals.

Fasting and water deprivation for 12 h within the 24-h period after molding, the rats were anesthetized using isoflurane (induction: 3–4%, maintenance: 2–2.5%), and then electrocardiogram detection was performed. If the electrocardiogram of the rats in the model group shows an upward shift of the J point and/or an ST segment deviation of ≥0.1 mv compared with that of the rats in the control group, it indicates that the myocardial ischemia model has been successfully established.

Following ethical euthanasia via cervical dislocation under deep anesthesia, the cardiac tissue was procured through a standardized protocol: Immediate thoracotomy with rapid heart excision, followed by three rinses in saline (0.9% NaCl) to remove residual blood. The left ventricular tissue was flash-frozen in liquid nitrogen within 90 s post-excision for cryopreservation at −80 °C, while the remaining cardiac specimen was immersion-fixed in 4% paraformaldehyde (PFA) dissolved in normal saline for 24 h at room temperature to prepare for histopathological processing.

A total of four rats died during the modeling period, among which three were from the model group and one was from the PTI-L group. Using the modeling outcomes, six rats per group were randomly selected for index measurement via a random number table generated by Microsoft Excel software.

#### 2.7.2. Electrocardiogram (ECG)

The animals were fixed on a rat plate, and respiratory anesthesia was induced using isoflurane. The leads were sequentially connected to the limbs of the rats and the ECG signals of each rat were recorded. The baseline ECG was recorded at the starting point of two consecutive QRS complexes, the J point of each group was determined, and the vertical distance from the J point to the baseline was measured using ImageJ 1.54d, which was recorded as the ST-segment offset value (mV). The length of each grid scale in the ECG paper was 0.02 ms, and the heart rate was obtained by multiplying the length of the grid-scale in the ECG paper by the number of cells (n) in the R-R interval. Heart rate = 60/(0.02 × n).

#### 2.7.3. Measurement of Serum Levels for IL-6, LDH, hs-CRP, cTnI, ROS, IL-1β, and TNF-α

Blood samples were collected in sterile blood collection vacuum tubes, and stored at ambient temperature for 120 min prior to centrifugation at 3500 rpm for 15 min at 4 °C using a refrigerated centrifuge. The serum supernatant was aliquoted, stored at −80 °C, and analyzed via commercial ELISA kits to quantify IL-6, LDH, hs-CRP, cTnI, ROS, IL-1β, and TNF-α levels following manufacturer protocols, with absorbance measured at 450 nm.

#### 2.7.4. Measurement of Tissue Levels for MDA and SOD

According to the references methodology [14], Cardiac tissue, disrupted and homogenized in a 1:9 saline solution, underwent centrifugation at 2500 rpm for 10 min; the resulting supernatant was then harvested. MDA levels and SOD activity in rat myocardial homogenates were quantified using a double-antibody sandwich ELISA kit (AiFang Biological, Cat# AF8503-A/AF3262-A, Changsha, China) following the manufacturer’s instructions.

#### 2.7.5. Hemorheology

Whole blood viscosity was measured using 1 mL of sodium citrate anticoagulant and an automatic flow meter. To measure the plasma viscosity, A 1-mL aliquot of citrated blood was processed through centrifugation (3000 rpm × 10 min, 4 °C).

#### 2.7.6. TUNEL Staining

Paraffin sections were dewaxed in xylene I/II/II (10 min each) and rehydrated through graded ethanol (100% I/II/III, 5 min each), followed by distilled water rinsing. Tissues were treated with Proteinase K working solution (20 μg/mL, 1:9 diluted in PBS) at 37 °C for 22 min, then washed in PBS (pH 7.4) on a shaker (3 × 5 min). Permeabilization with 0.1% Triton (1:1000 in PBS) was performed at RT for 20 min, followed by PBS washes. TUNEL reaction mix (TdT enzyme:dUTP = 1:50) was applied and incubated at 37 °C (1–2 h, dark). After PBS washes, nuclei were counterstained with DAPI (10 min, dark), and slides were mounted with the anti-fade medium. Apoptotic cells were visualized under a fluorescence microscope: DAPI for nuclei, and TUNEL-positive cells were quantified for apoptosis rate.

#### 2.7.7. Wb of Apoptosis-Related Effectors in Cardiac Tissue

To initiate the protocol, we extracted 15 to 20 mg of the left ventricular apex and blended it with 200 μL of RIPA lysis buffer, which was enhanced with a 1% protease inhibitor mix. Once we let the mixture sit at room temperature (RT) for a solid 1 to 2 min, we spun it down at 14,000 to 16,000× *g* for another quick 1 to 2 min to collect the supernatant. Next, We used a BCA assay to determine the protein concentration. Then, we denatured 30 μg of each batch in 5× Loading Buffer by zapping it at 95 °C for a full five min. Those specimens were then loaded onto 10% SDS-PAGE gels and zapped with electricity (stacking gel: 80 V for half an hour; resolving gel: 120 V for 90 min), followed by transfer to 0.45 μm PVDF membranes using a wet transfer method (200 mA for 90 min). To prevent any accidental bonding, we soaked the membranes in 5% skim milk with TBST for a good hour at room temp. Post-blocking, we left the membranes with primary antibodies targeting Bax, Bcl-2, Caspase-3, and Caspase-9 (diluted 1:1000) overnight in the fridge, followed by a brief 20-min sit at RT. The membranes were cleaned with TBST (three times, each for 5 min), then treated with an HRP-conjugated secondary antibody (1:10,000) for an hour at 37 °C. After a few more TBST rinses (four times, 5 min each), we visualized the protein bands and employed ImageJ software to assess the intensity of the bands, computing the ratio of the target protein to GAPDH.

#### 2.7.8. Myocardial Pathological Changes Were Measured Using HE Staining

Three rats per group were used. Hearts were fixed in 4% paraformaldehyde, dehydrated through graded ethanol (75%, 80%, 95%, 100%), cleared in xylene, embedded in paraffin, and microtomed to 4 μm (3 consecutive sections per heart). Tissue sections were processed by hematoxylin staining following dewaxing (5 min) differentiated in acid ethanol, counterstained with eosin (2 min), and mounted with neutral gum. Histopathological scoring criteria [15]: Score 0: no tissue damage; Score 1 (mild): interstitial edema alongside focal necrotic foci; Score 2 (moderate): widespread myocardial swelling and necrosis; Score 3 (severe): contraction band necrosis with neutrophil infiltration and interstitial collagenization; Score 4 (maximal severity): diffuse contraction band necrosis, neutrophil infiltration, interstitial collagen deposition, and hemorrhage. Six non-overlapping fields per section (×200, covering ischemic, border, and normal zones) were analyzed, yielding 54 images per group (3 rats × 3 sections × 6 fields). Results were averaged.

#### 2.7.9. Masson’s Trichrome Staining

Three rats per group were used. Following 24-h fixation in 4% paraformaldehyde, heart samples were embedded in paraffin and cut horizontally at the apical level to obtain three consecutive 4-μm sections per heart. Post-xylene dewaxing and sequential ethanol rehydration, the tissue slices were subjected to coloring. Sections underwent sequential staining: 5-min iron hematoxylin staining, 10-min Biebrich scarlet-acid fuchsin staining, 5-min phosphomolybdic acid treatment, and 5-min aniline blue staining. They were then differentiated in 1% acetic acid, dehydrated, xylene-cleared, and mounted with neutral gum. Collagen deposition was quantified as the percentage of the blue-stained fibrotic area using ImageJ. Six non-overlapping fields per section (×200, covering the ischemic core, border zone, and normal myocardium) were analyzed, yielding 54 images per group (3 rats × 3 sections × 6 fields).

#### 2.7.10. Statistical Analysis

Statistical analyses were performed with GraphPad Prism 8.0. Data normality was determined through Shapiro-Wilk testing (n ≤ 50) or Kolmogorov-Smirnov testing (n > 50). Parametric intergroup comparisons employed one-way ANOVA with Tukey’s post hoc analysis, whereas nonparametric datasets underwent Kruskal-Wallis testing with subsequent Dunn’s multiple comparison adjustment. For dual-group evaluations, the Student’s *t*-test or Mann-Whitney U-test was selectively applied based on distribution characteristics. Quantitative data are expressed as means ± SD, with statistical thresholds set at *p* < 0.05 (two-tailed).

## 3. Results

### 3.1. UPLC-Q-TOF/MS

**Figure 1:**
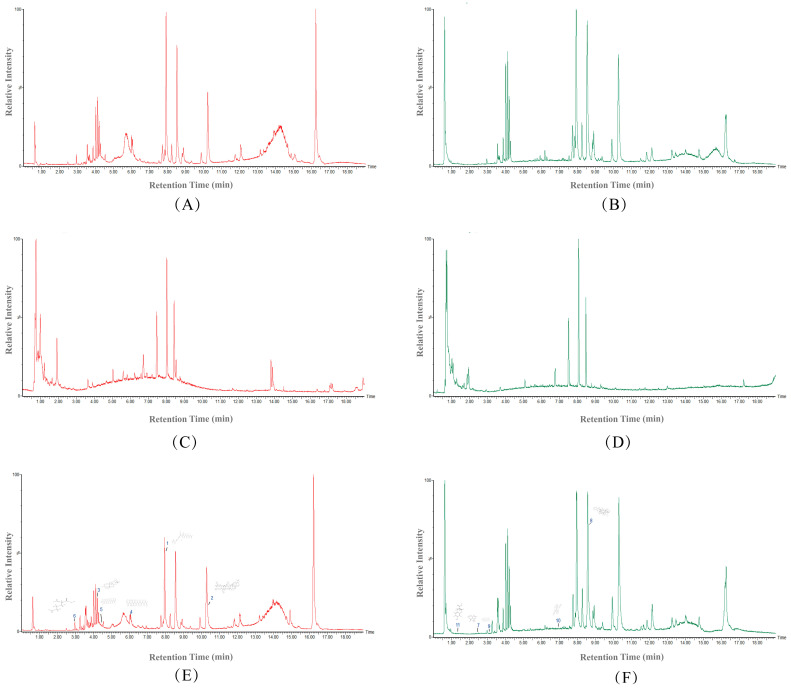
Total ion chromatograms. (**A**) ESI+ analysis of blank serum matrix; (**B**) ESI- analysis of blank serum matrix; (**C**) PTI chemical fingerprint in ESI+ mode; (**D**) PTI chemical fingerprint in ESI− mode; (**E**) Drug-containing serum metabolic profile (ESI+); (**F**) Drug-containing serum metabolic profile (ESI−). Numbers 1–11 represent “Components identified” in Table 1.

Biological samples underwent qualitative analysis via UPLC-Q-TOF/MS. Shown in Figure 1 are the total ion chromatograms (TIC) of the samples acquired in ESI+ and ESI−. There are 11 active ingredients in blood serum, of which six are in ESI+ of medicine serum (Figure 1E) and five are in ESI− of medicine serum (Figure 1F).

### 3.2. Hemal Constituents Profiling and Serum Pharmacochemistry of PTI Intervention

**Figure 2:**
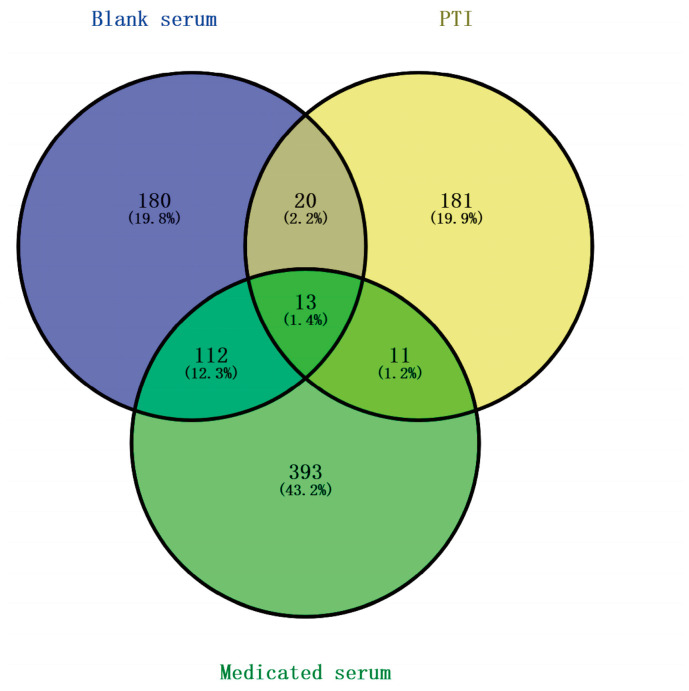
Venn Diagram Illustrating PTI’s Active Blood Compounds.

The blank group consisted of 325 active ingredients, the PTI group consisted of 225 active ingredients and the medicated serum group consisted of 529 active ingredients. There were 11 active ingredients of blood serum, which were composed of the active ingredients of the PTI group and the active ingredients of the medicated serum group to remove the active ingredients of the blank group. (Figure 2).

### 3.3. Characterization of Absorbed Blood Components

Post-identification, the PTI sample comprised 11 distinct blood components (Table 1).

**Table 1:** Components identified in PTI by UPLC-Q-TOF/MS.

No.	tR/min	Identification	Formula	Selected Ion	Expected	Detected	M/Z	Error (ppm)	Fragment Ion MS2
1	7.96	Ciryneol C	C_16_H_23_ClO_2_	[M + Li]+	282.1387	282.1391	289.1545	1.49	184.0861; 131.0951; 109.1081
2	10.41	Cynanoside M	C_38_H_56_O_13_	[M + NH_4_]+	720.3720	720.3687	738.4028	−4.22	497.3144
3	4.28	Darutigenol	C_20_H_34_O_3_	[M + NH_4_]+	322.2507	322.2491	340.2828959	−5.3	114.0986; 133.0945; 96.0864
4	6.06	Hexadecane	C_16_H_32_	[M + Li]+	224.2504	224.2510	231.2664	2.45	102.0976
5	4.42	n-Tetradecaal	C_14_H_28_O	[M + Li]+	212.2140	212.2134	219.2289	−2.72	114.0987; 133.0956
6	2.96	Ornithine	C_5_H_12_N_2_O_2_	[M + e]+	132.0899	132.0895	132.0890	−2.78	115.0615; 103.0602
7	2.49	Gentiatibetine	C_9_H_11_NO_2_	[M-H]−	165.0790	165.0783	164.0710	−4.19	151.8964
8	8.93	Hokbusine A	C_32_H_45_NO_10_	[M-CI]−	603.3075	603.3043	638.2769	4.90	396.1784; 224.0686
9	3.11	n-Butyl-α-D-fructopyranoside	C_10_H_20_O_6_	[M-CH_3_COO]−	236.1260	236.1261	295.1400	0.44	194.9491
10	6.93	Pseudoaspidin	C_25_H_32_O_8_	[M-H]−	460.2097	460.2085	459.2012	−2.68	196.8942
11	1.35	Tyrosine	C_9_H_11_NO_3_	[M-H]−	181.0739	181.0732	180.0659	−3.73	78.9571

### 3.4. Construction of the PTI Targets, Acute Myocardial Ischemia-Related Targets, and the Common Targets of PTI-Acute Myocardial Ischemia

**Figure 3:**
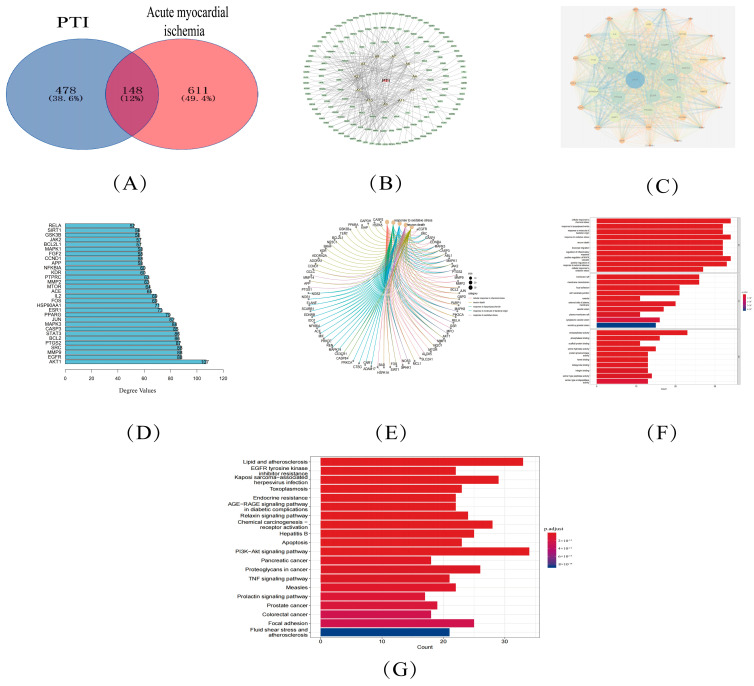
Network pharmacology-based therapeutic mechanism prediction of PTI for acute myocardial ischemia. (**A**) Venn diagram of shared targets between PTI and disease (Blue: PTI targets; Red: Acute myocardial ischemia targets; Overlap: Common targets). (**B**) “PTI-active component-target” interaction network (Red: PTI; Yellow: Active ingredients; Green: Common targets). (**C**) PPI network of common targets (Degree ≥ 50). Node color and size gradients: Darker blue with larger area (high degree); Darker red with smaller area (low degree). Edge thickness and color: blue lines (thicker for higher combined scores); red lines (thinner for lower combined scores). (**D**) Top 30 core targets ranked by degree value. (**E**) Biological process (BP)-target relationship mapping. (**F**) GO analysis results. (**G**) KEGG pathway analysis results.

In total, 745 acute myocardial ischemia targets were chosen from the Genecards database and 32 selected objectives originated within DisGeNET. The integrated disease databases underwent deduplication, yielding 760 unique disease-associated target genes. PERL software(5.30.0.1) was screened to generate 148 common targets of PTI-acute myocardial ischemia, and the results were visualized using a VENNY plot (Figure 3A).

### 3.5. PTI -Core Component-Common Target

Core components were chosen by network analysis centrality., revealing that the key targets of PTI in treating acute myocardial ischemia included pseudoaspidin, ciryneol C, cynanoside M, darutigenol, and n-butyl-β-d-fructofuranoside. Figure 3B shows the “PTI-active component-common target” network diagram (Table 2).

### 3.6. PPI Network Analysis

Topological interrogation of the protein-protein interaction (PPI) network revealed an architecture comprising 148 protein nodes interconnected through 2503 interaction edges. Network metrics demonstrated substantial connectivity patterns, with a mean nodal connectivity of 33.8 and a mean local clustering coefficient of 0.569, indicative of modular organization. Visualization of this interactome architecture is presented in Figure 3C.

Bar graphs depicting the top 30 target genes were visualized using R X64 4.0.4. Core targets were identified by prioritizing nodes with high degree values in the network analysis, revealing that the key targets of PTI for acute myocardial ischemia were AKT 1, EGFR, MMP 9, and SRC (Figure 3D).

### 3.7. GO and KEGG Analysis

A total of 2521 biological process (BP) terms were identified through GO enrichment analysis. There were 106 entries for cell components (CC) and 204 entries for molecular function (MF).

Figure 3E presents the interactions between BP-related targets, while Figure 3F displays the GO enrichment analysis results.

There were 171 KEGG biological pathways, including disease pathways, such as the PI3K-Akt signaling pathway and lipid and atherosclerosis (Figure 3G).

### 3.8. Results of Molecular Docking

**Figure 4:**
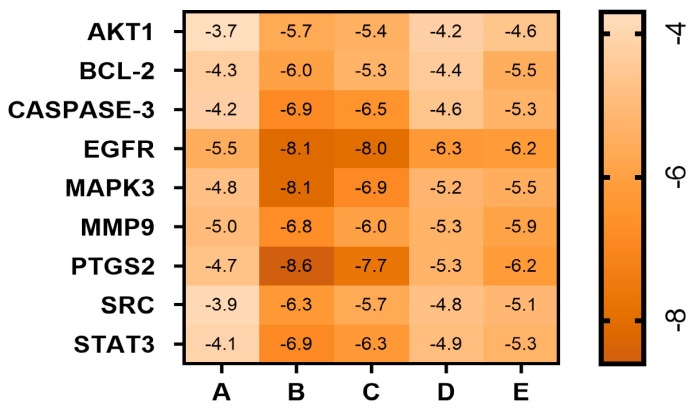
Heatmap of PTI molecular docking with key targets. A: ciryneol C; B: cynanoside M; C: darutigenol; D: n-butyl-β-D-fructopyranoside; E: Pseudoaspidin.

**Figure 5:**
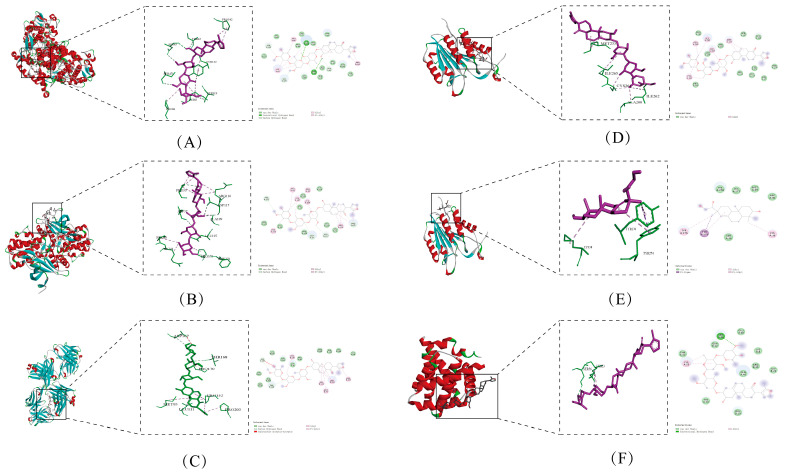
Results of molecular docking diagram (**A**) PTGS2-Cynanoside M (**B**) MAPK3-Cynanoside M (**C**) EGFR-Cynanoside M (**D**) CASPASE3-Cynanoside M (**E**) CASPASE3-Darutigenol (**F**) BCL2-Cynanoside M. Red represents α-helix, green represents loop, and blue represents β-sheet.

The molecular docking analysis of the top 10 target proteins and 5 active ingredients in the PPI network map revealed that PTGS 2-cynanoside M had the absolute value highest docking score (−8.6 kcal/mol), followed by MAPK 3-cynanoside M (−8.1 kcal/mol), EGFR-cynanoside M (−8.1 kcal/mol), CASPASE3-Cynanoside M (−6.9 kcal/mol), CASPASE3-Darutigenol (−6.5 kcal/mol) and BCL2-Cynanoside M (−6.0 kcal/mol). PTGS2-Cynanoside M generated conventional hydrogen bonds at TYR122 and ASN43 sites in the 3olt conformation. MAPK3-Cynanoside M generated carbon-hydrogen bonds at the PHE371, ASP37, ASP117, and PRO35 sites in the 2zoq conformation. EGFR-cynanoside M generated carbon-hydrogen bonds at the ASP167 and THR168 sites in the 2xkn conformation. Cynanoside M forms alkyl bonds with Met233 and Ile265 in Caspase-3; darutigenol interacts with Tyr274 in Caspase-3 via π-σ bonds; cynanoside M creates a hydrogen bond with Ser56 in Bcl-2. These results suggested that the molecular docking of active compounds with their corresponding targets exhibited strong binding affinity. Molecular docking outcomes between active components in blood samples and target proteins are presented in Figure 4 and Figure 5. We performed the molecular docking three times, calculated the mean and standard deviation, and uploaded the results in the Supplementary Materials (Table S1).

### 3.9. The Results of ECG

**Figure 6:**
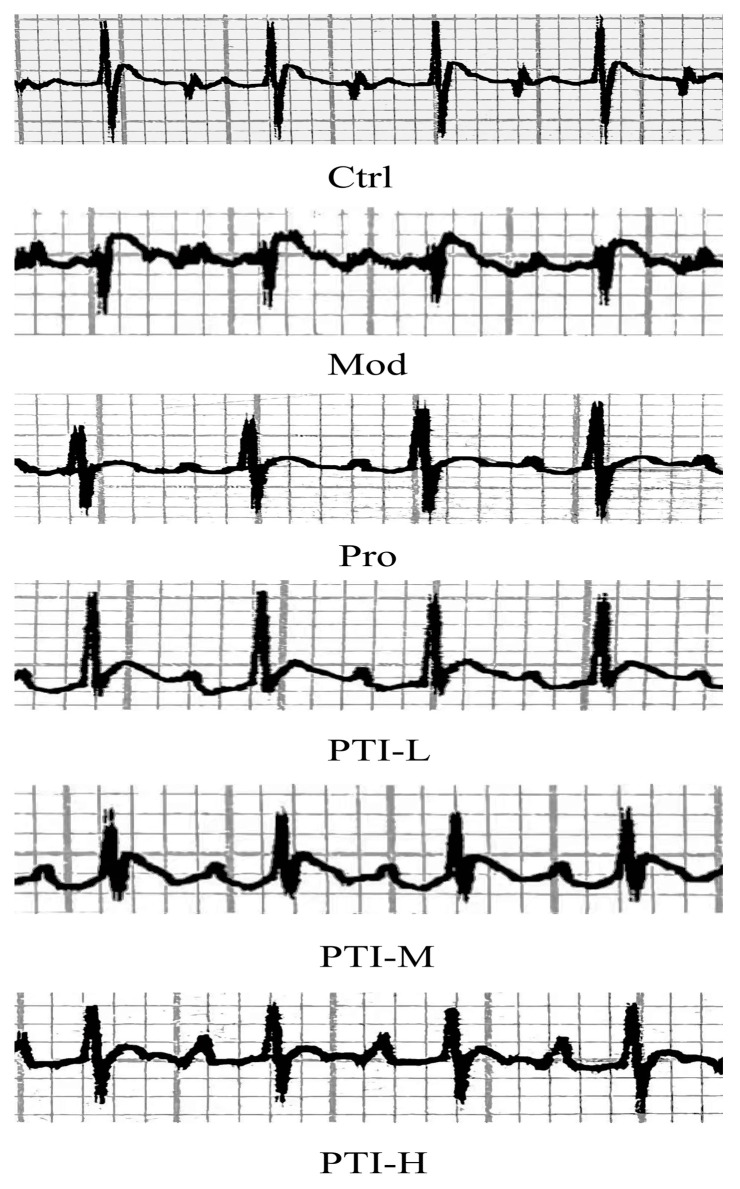
Effect of PTI on ECG in rats with acute myocardial ischemia (n = 6). Ctrl-control group, Mod-model group, Pro- propranolol group, PTI-L-PTI low-dose group, PTI-M-PTI medium-dose group, PTI-H-PTI high-dose group.

Unlike the control group, some model rats exhibited an increased heart rate (*p* < 0.01), increased ST offset (*p* < 0.01), T-wave elevation, and even inversion (Table 3 and Figure 6). These findings demonstrated the successful preparation of the myocardial ischemia. Compared to the model group, PTI-L, PTI-M, PTI-H, and Pro groups reduced ST offset (*p* < 0.01). Pro and PTI-H group showed the most pronounced effects (*p* < 0.05), but heart rate remained unchanged without statistical significance (*p* > 0.05).

### 3.10. Results of HE Staining

**Figure 7:**
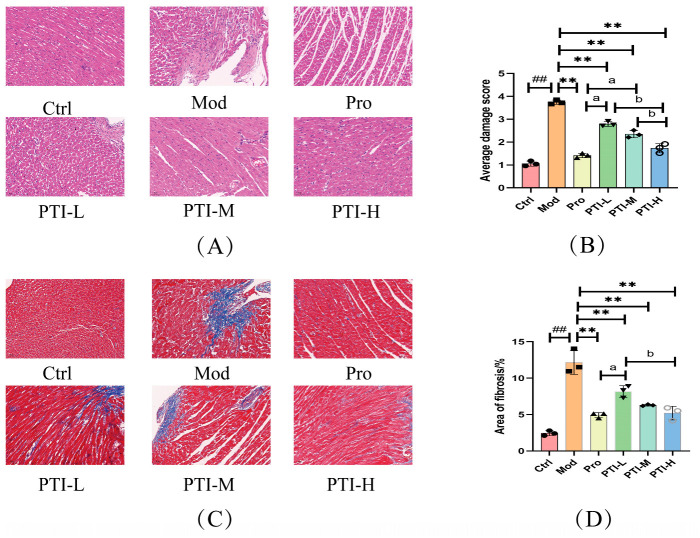
Influence of PTI on myocardial pathology triggered by ISO in rats suffering from acute myocardial ischemia. (**A**) HE staining (n = 3) (magnification, 200×); (**B**) Average damage score (**C**) Masson staining (n = 3) (magnification, 200×). (**D**) Fibrinogen deposition rate. Results presented as mean ± SD (n = 3); statistical significance marked with ^##^ *p* < 0.01 vs. control and ** *p* < 0.01 vs. model. ^a^ *p* < 0.05 vs. Pro group, ^b^ *p* < 0.05 vs. PTI-H group. Ctrl-control group, Mod-model group, Pro- propranolol group, PTI-L-PTI low-dose group, PTI-M-PTI medium-dose group, PTI-H-PTI high-dose group.

As illustrated in Figure 7, control group cardiomyocytes maintained normal structural morphology: cardiac muscle fibers were regularly arranged, uniformly stained, and maintained a typical intercellular space without structural abnormalities. No obvious lymphocyte infiltration, vascular lumen expansion, or congestion was observed in the interstitium. Among model group animals, severe hypertrophy of myocardial fibers, severe widening of the myocyte space, obvious collagenization, massive lymphocyte infiltration, obvious expansion, congestion, and bleeding were observed in the interstitium. In the propranolol group, cardiac myocytes exhibited a slight increase in size, whereas the muscle fibers showed a slight lack of organization. The cardiac muscle fibers were slightly thicker, and tissue staining was slightly uneven. Additionally, the space between myocytes was slightly wider. The myocardial tissue showed slight signs of collagen deposition. Lymphocyte infiltration was observed in the interstitium and the vascular lumen showed slight dilation, congestion, and bleeding. Furthermore, PTI-L myocytes exhibited a moderate increase in size and moderate enlargement of muscle fibers, whereas PTI-M myocytes showed greater infiltration of lymphocytes into the surrounding tissue. The PTI-H group exhibited cardiomyocytes with slight hypertrophy, a mild arrangement of muscle fibers, and slight enlargement of the space between myocytes. Additionally, mild collagenization of the myocardial tissue and small lymphocyte infiltration in the interstitium were observed. Mild expansion of the vascular cavities, congestion, and bleeding were also observed in this group. Myocardial injury was substantially worsened in the model group (*p* < 0.01). In contrast, propranolol (Pro), PTI-L, PTI-M, and PTI-H treatments markedly attenuated myocardial injury (*p* < 0.01). Pro and PTI-H groups showed the most pronounced effects (*p* < 0.05).

### 3.11. Masson’s Trichrome Staining Results

As illustrated in Figure 7, Masson’s trichrome staining was employed to detect collagen fibers within muscle tissue. This method enables the visualization of collagen fibers by staining them blue and red, thereby differentiating between collagen and muscle fibers. No significant collagen fiber deposition was noted in the control group samples. In the model group, however, a notable elevation in collagen content was observed. A trace of collagen deposition was observed in the propranolol-treated group. In contrast, the PTI-M and PTI groups exhibited mild collagenization, while the PTI-L group showed moderate collagenization (*p* < 0.01).

### 3.12. PTI Attenuates Myocardial Ischemia Biomarkers: Inflammatory, Cardiac, and Oxidative Mediators in Rat Serum

**Figure 8:**
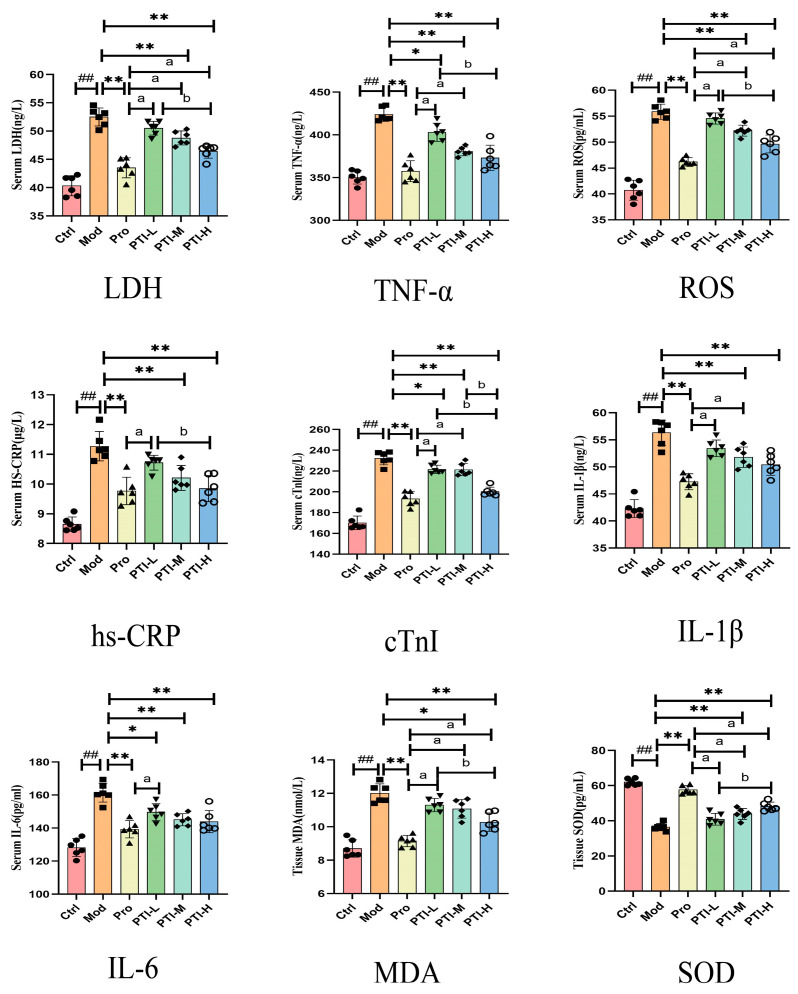
Levels of IL-6, LDH, hs-CRP, cTnI, ROS, IL-1β, TNF-α, MDA, and SOD. Results presented as mean ± SD (n = 6); statistical significance marked with ^##^ *p* < 0.01 vs. control, ** *p* < 0.01 and * *p* < 0.05 vs. model. ^a^ *p* < 0.05 vs. Pro group, ^b^ *p* < 0.05 vs. PTI-H groupCtrl- control group, Mod-model group, Pro- propranolol group, PTI-L-PTI low-dose group, PTI-M-PTI medium-dose group, PTI-H-PTI high-dose group.

As illustrated in Figure 8, Biochemical profiling demonstrated a significant elevation of serum inflammatory mediators (IL-6, IL-1β, TNF-α), cardiac necrosis indicators (cTnI, LDH), and oxidative stress markers (ROS, hs-CRP) in acute myocardial ischemia model rats versus controls (*p* < 0.01). Therapeutic interventions exhibited dose-dependent efficacy: both high-dose PTI (PTI-H) and propranolol significantly attenuated all biomarkers (*p* < 0.01), while medium-dose PTI (PTI-M) effectively reduced IL-6, TNF-α, LDH, ROS, and hs-CRP (*p* < 0.01). Low-dose PTI (PTI-L) showed partial modulation of IL-6, TNF-α, and cTnI (*p* < 0.05), with nonsignificant effects on IL-1β, ROS, LDH, and hs-CRP (*p* > 0.05). Pro and PTI-H groups showed the most pronounced effects (*p* < 0.05).

### 3.13. PTI Attenuates Myocardial Ischemia Biomarkers: Oxidative Mediators in Rat Tissue

As illustrated in Figure 8, Oxidative stress evaluation revealed compromised antioxidant capacity in myocardial ischemia models, with significantly depressed superoxide dismutase (SOD) activity (*p* < 0.01) and elevated malondialdehyde (MDA) levels (*p* < 0.01) versus controls. Therapeutic regimens demonstrated differential redox modulation: Propranolol, PTI-M, and PTI-H significantly attenuated lipid peroxidation (MDA reduction, *p* < 0.01) but failed to restore superoxide dismutase (SOD) activity (*p* > 0.05). Pro and PTI-H groups showed the most pronounced effects (*p* < 0.05).

### 3.14. PTI-Mediated Modulation of Cardiomyocyte Apoptosis in Acute Myocardial Ischemia Rats

**Figure 9:**
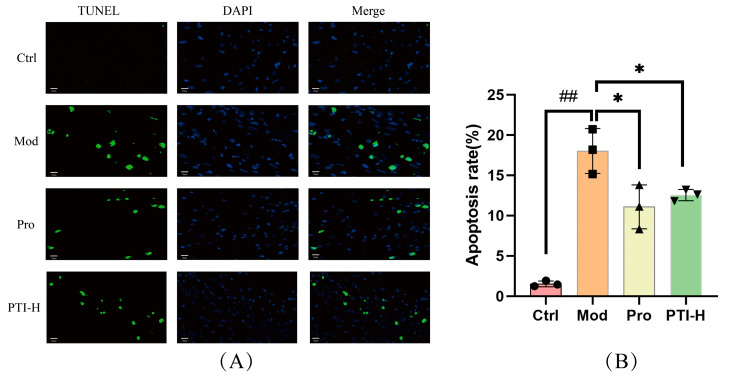
Effect of PTI on cardiomyocyte apoptosis in the rat model of acute myocardial ischemia. (**A**) TUNEL assay (magnification, 400×); (**B**) Apoptosis rate. Results presented as mean ± SD (n = 6); statistical significance marked with ^##^ *p* < 0.01 vs. control and * *p* < 0.05 vs. model. Ctrl- control group. Mod-model group, Pro- propranolol group, PTI-H-PTI high-dose group.

Cardiomyocyte apoptosis quantification demonstrated significantly elevated TUNEL+ cell density in myocardial ischemia models versus controls (*p* < 0.01). Pharmacological interventions with propranolol and high-dose PTI (PTI-H) exhibited marked attenuation of the apoptotic index compared to the model group (*p* < 0.05) (Figure 9).

### 3.15. Effect of PTI on Blood Rheology in Acute Myocardial Ischemia Rats

Model rats exhibited a marked increase in whole blood and plasma viscosity (*p* < 0.01). Propranolol administration significantly lowered these values (*p* < 0.01), whereas PTI-H treatment resulted in a notable reduction in both viscosity parameters (*p* < 0.05) (Table 4).

### 3.16. PTI-Mediated Regulation of Bax, Bcl-2, Caspase-3, and Caspase-9 Expression in Acute Myocardial Ischemic Rat Hearts

**Figure 10:**
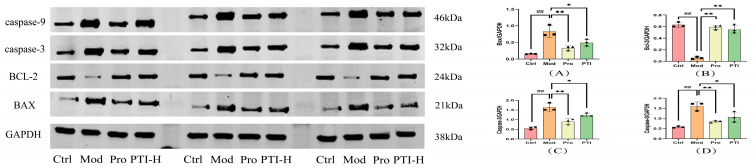
PTI influences the expression of pro-apoptotic and anti-apoptotic proteins in ischemic rat myocardium. (**A**) Western blotting for BAX, (**B**) BCL-2, (**C**) caspase-3, and (**D**) caspase-9. Results presented as mean ± SD (n = 6); statistical significance marked with ^##^ *p* < 0.01 vs. control, ** *p* < 0.01 and * *p* < 0.05 vs. model. Ctrl- control group, Mod-model group, Pro- propranolol group, PTI-H-PTI high-dose group. Original images can be found in Figure S1.

The model group showed a marked increase in pro-apoptotic proteins (Bax, Caspase-3, Caspase-9) (*p* < 0.01) and a significant decrease in anti-apoptotic Bcl-2 (*p* < 0.01) compared with the control group. In contrast to the model group, both treatment groups (propranolol and PTI-H) displayed modest decreases in pro-apoptotic marker expression (*p* < 0.05) and a notable increase in Bcl-2 levels (*p* < 0.01), as shown in Figure 10.

## 4. Discussion

Acute myocardial ischemia represents a pathological state arising from insufficient oxygen and blood supply to the heart. This results in abnormal energy metabolism in heart muscle cells (cardiomyocytes), leading to an inability to maintain normal heart function.

UPLC-Q-TOF/MS enables the accurate identification of pharmacological components in drugs, furnishing reliable data rooted in chemical substances for network pharmacology analyses. Based on these data, network pharmacology predicts drug targets and related pathways, and molecular docking is used to conduct microscopic interaction verification and detailed exploration of key targets and active ingredients predicted by network pharmacology. In this way, from macro to micro, from material basis to the mechanism of action, the process of action of drugs in living organisms is comprehensively and deeply analyzed, which makes up for the shortcomings of a single technology in mechanism analysis. This results in an abnormal energy metabolism in cardiomyocytes, making it impossible to maintain normal heart function [16]. Cynanoside M is a flavonoid with antioxidant properties. Studies have demonstrated that cynanosides effectively preserve mitochondrial function and prevent cell death by decreasing ROS generation [17]. The study revealed that darutigenol, a diterpenoid compound with potent anti-inflammatory effects, effectively suppresses the release of inflammatory mediators while accelerating the clearance of ROS and nitric oxide (NO) [18].

Potential targets related to CHD were identified through KEGG pathway enrichment analysis, which included key pathways like the PI3K/Akt signaling cascade and EGFR tyrosine kinase inhibitor resistance mechanism. The PI3K/Akt signaling pathway represents a key intracellular signaling cascade that mediates responses to extracellular cues, regulating critical processes such as cellular metabolism, proliferation, growth, and angiogenesis. Investigations have revealed that the PI3K/Akt signaling pathway is closely associated with apoptosis and can inhibit apoptosis to ameliorate myocardial injury after acute myocardial ischemia [19]. Akt is among the most important targets in this signaling pathway. Detrimental members of the Bcl-2 family can bind to Bcl-2, thereby inhibiting its anti-apoptotic activity. Additionally, it has been demonstrated that the phosphorylation of Bad by Akt enhances the anti-cell death effects of Bcl-2. Simultaneously, phosphorylated Akt inhibits the release of mitochondrial cytochrome C, deactivates Caspase-9 phosphorylation, hinders the formation of apoptotic bodies, and subsequently affects the activation of Caspase-3. In the presence of extracellular signals, PI3K activates Akt to promote its phosphorylation. Phosphorylated Akt acts on downstream substrates that contain serine/threonine residues, thereby exerting anti-apoptotic effects [20].

PPI network analysis identified AKT 1, EGFR, MMP 9, SRC, PTGS 2, STAT 3, BCL 2, CASP 3, and MAPK 3 as the key targets of PTI in the treatment of acute myocardial ischemia. The primary involvement of these targets lies in inflammation, myocardial fibrosis, and apoptosis. PTGS 2, also known as COX-2, is a bifunctional enzyme that contributes to the inflammatory response by acting as both catalase and cyclooxygenase. Investigations have indicated that suppression of COX-2 expression effectively mitigates myocardial ischemia and relieves coronary artery spasms during acute ischemic events [21,22]. Bax is a constituent of the Bcl protein family that promotes apoptosis, while Caspase-3 is a cysteinyl protease necessary for triggering neuronal apoptosis. Evidence has shown that inhibition of Caspase-3 expression effectively attenuates both apoptotic and necrotic cell death pathways, thus presenting a promising therapeutic strategy for ischemic injury [23]. Moreover, Bcl-2 hinders apoptosis by eliminating ROS, whereas Bax hampers the activity of Bcl-2, leading to apoptosis. Bax has been shown to upregulate Bcl-2 expression; conversely, downregulating Bax expression can inhibit apoptosis and alleviate myocardial ischemia [24].

Based on molecular docking results, our study revealed that CASPASE3 and BCL2—two key genes in the apoptotic pathway—exhibit strong binding affinities with active components of PTI, such as Cynanoside M and Darutigenol, suggesting a close association between PTI and apoptotic regulation. Notably, Cynanoside M displayed the highest binding affinities with PTGS2, MAPK3, and EGFR in molecular docking analyses. These could potentially be ideal focal points for subsequent studies on the expanded dynamics of PTI.

Subcutaneous injection of high doses of ISO overexcites cardiac β1 receptors, significantly increasing myocardial oxygen consumption and leading to the development of acute myocardial ischemia [25]. After administering 85 mg/kg ISO from Day 6, ECG showed an elevated ST segment and increased heart rate. These findings suggest the occurrence of acute myocardial ischemia in rats, consistent with results from prior studies [26].

IL-6 is a primary inflammatory factor whose expression is highly increased in patients with acute myocardial ischemia. This increases the inflammatory response and exacerbates myocardial ischemia [27]. LDH is a myocardial enzyme in the energy metabolism of cardiomyocytes and its level is an important indicator of myocardial ischemia [28]. Studies have shown that abnormal increases in LDH levels can stimulate myocardial anaerobic metabolism and aggravate cell damage [29]. Reactive oxygen species (ROS) mediate oxidative modification of critical biomolecules—including but not limited to lipid membranes, protein complexes, and nucleic acid polymers—through free radical interactions that compromise structural integrity and physiological activity. These molecular-level disruptions initiate oxidative damage cascades, ultimately culminating in necrotic pathway activation and irreversible cellular demise. As a prominent inflammatory cytokine, IL-1β contributes significantly to the development of cardiovascular disease [30]. IL-1β can lead to fiber atherosclerosis, and plaque injury, and reduce inflammatory responses [31]. TNF-α is a major inflammatory factor. Studies have reported that High levels of TNF-α can cause myocardial hyperplasia, fibrosis, cardiomyocyte apoptosis, and vascular endothelial cell damage. This can aggravate myocardial ischemia by thickening the intima and inducing cell damage [32]. CRP is a marker of the inflammatory response that is highly expressed in the endothelial cells of patients with myocardial ischemia. CRP can inhibit angiogenesis in coronary plaques and compromise the structural integrity of the blood vessels. Therefore, low CRP levels can improve myocardial ischemia management [33]. cTnI is an effective indicator for the diagnosis of acute myocardial ischemia [34]. As rats with acute myocardial ischemia exhibit a substantial increase in cTnI levels, low levels of cTnI indicate sufficient management of cardiomyocyte injury [35]. SOD is a metalloenzyme with strong oxygen radical scavenging ability. Investigations have indicated that enhanced superoxide dismutase (SOD) activity potently alleviates myocardial injury following the onset of acute myocardial ischemia [36]. MDA serves as a result of ROS-induced oxidation, offering a measure of lipid peroxidation intensity in vivo and suggesting cellular damage levels [37].

ECG is a highly effective tool for diagnosing acute myocardial ischemia [38]. Our findings showed a statistically significant increase in ST-segment offset values in the model group (*p* < 0.01), confirming the successful induction of acute myocardial ischemia in rats. TUNEL staining revealed a higher proportion of apoptotic cardiomyocytes in model rats compared to controls. PTI-H treatment led to a marked decrease in apoptosis rates versus the model group. Taken together, these results demonstrate that PTI attenuates myocardial tissue injury and suppresses apoptosis following ischemic stress.

Myocardial ischemia is closely associated with haematological abnormalities. Increased blood viscosity leads to increased blood flow resistance and a decreased flow rate. This can greatly reduce the blood flow in the coronary arteries and trigger or worsen myocardial ischemia. Our findings demonstrated PTI can significantly decrease the abnormally elevated viscosity of both whole blood and plasma following myocardial ischemia.

## 5. Conclusions

This study employed a multidisciplinary approach combined UPLC-Q-TOF/MS analysis, network pharmacology, molecular docking studies, and animal trials in vivo to illustrate that Pericarpium Trichosanthis injection (PTI) alleviates acute myocardial ischemia by inflammation and apoptosis, with core active components (e.g., cynanoside M) demonstrating strong binding affinity to targets such as PTGS2, MAPK3, EGFR, CASPASE-3, and BCL-2. It should be noted that although the ISO-induced rat model is widely used to simulate acute myocardial ischemia, further validation using multiple models is still necessary in the future. Additionally, based on the findings of this study, we will further investigate the mechanism by which PTI regulates apoptosis via the PI3K/Akt pathway, aiming to provide new insights and directions for the treatment of acute myocardial ischemia with TCM.

## Figures and Tables

**Table 2:** The core ingredients.

Degree	ID	Name	Average Shortest Path Length	Betweenness Centrality
41	A10	Pseudoaspidin	2.42138365	0.24892873
34	A1	Ciryneol C	2.50943396	0.18568535
30	A2	Cynanoside M	2.55974843	0.1418833
29	A3	Darutigenol	2.57232704	0.12863103
29	A7	n-Butyl-β-D-fructopyranoside	2.57232704	0.14833691

**Table 3:** ST Segment deviation and heart rate.

Group	ST Segment Deviation/(×10^3^)/mV	Heart Rate/(bp·min^−1^)
Ctrl	25 ± 3.61	389.58 ± 31.66
Mod	124.33 ± 17.9 ^##^	557.61 ± 16.91 ^##^
Pro	30.33 ± 7.51 **	503.4 ± 21.33
PTI-L	65 ± 10.82 **^,a,b^	510.58 ± 74.05
PTI-M	51.33 ± 3.21 **	530.02 ± 40.69
PTI-H	37 ± 2 **	539.51 ± 21.77

Note: Results presented as mean ± SD (n = 6); statistical significance marked with ^##^ *p* < 0.01 vs. control and ** *p* < 0.01 vs. model. ^a^ *p* < 0.05 vs. Pro group, ^b^ *p* < 0.05 vs. PTI-H group.

**Table 4:** Effect of PTI on hemorheology in rats with acute myocardial ischemia.

Group	Whole Blood Viscosity (mPa·S)			Plasma Viscosity (mPa·S)
	Low Cut (1/5)	Medium Cut (1/50)	High Cut (1/200)	
Ctrl	11.21 ± 1.28	5.55 ± 0.73	4.3 ± 0.31	1.29 ± 0.05
Mod	19.32 ± 1.31 ^##^	9.24 ± 0.8 ^##^	5.3 ± 0.48 ^##^	1.83 ± 0.09 ^##^
Pro	13.13 ± 0.87 **	7.3 ± 0.83 **	4.11 ± 0.3 **	1.46 ± 0.06 **
PTI-H	14.3 ± 1.29 **	8.28 ± 0.54	3.89 ± 0.57 **	1.61 ± 0.16 *

Note: Results presented as mean ± SD (n = 6); statistical significance marked with ^##^ *p* < 0.01 vs. control, ** *p* < 0.01 and * *p* < 0.05 vs. model.

## Data Availability

Experimental datasets generated during this investigation are accessible through formal correspondence with the corresponding author (Hu, Z.X.).

## Supplementary Materials

The following supporting information can be downloaded at: https://www.mdpi.com/article/10.3390/biom15050618/s1, Figure S1: Original Western blot images; Table S1: The 3 times Molecular docking binding energies results (kcal/mol).

## References

[1] Shah M., Patil S., Patel B., Agarwal M., Davila C.D., Garg L., Agrawal S., Kapur N.K., Jorde U.P. (2018). Causes and Predictors of 30-Day Readmission in Patients with Acute Myocardial Infarction and Cardiogenic Shock. Circ. Heart Fail..

[2] Zhao D., Liu J., Wang M., Zhang X., Zhou M. (2019). Epidemiology of cardiovascular disease in China: Current features and implications. Nat. Rev. Cardiol..

[3] Xu F., Li L., Wang S., Zhao B., Li L. (2023). Effects of Hua Tuo Heart-Saving Pill on isoprenaline-induced acute myocardial ischemia in mice. Chin. Tradit. Pat. Med..

[4] Li H., Zhang S., Li F., Qin L. (2016). NLRX1 attenuates apoptosis and inflammatory responses in myocardial ischemia by inhibiting MAVS-dependent NLRP3 inflammasome activation. Mol. Immunol..

[5] Rischpler C. (2016). Acute myocardial infarction. Q. J. Nucl. Med. Mol. Imaging.

[6] Khan O., Patel M., Tomdio A.N., Beall J., Jovin I.S. (2023). Beta-Blockers in the Prevention and Treatment of Ischemic Heart Disease: Evidence and Clinical Practice. Heart Views.

[7] Loth D.W., Brusselle G.G., Lahousse L., Hofman A., Leufkens H.G.M., Stricker B.H. (2014). β-adrenoceptor blockers and pulmonary function in the general population: The Rotterdam Study. Br. J. Clin. Pharmacol..

[8] Yang L., Liu X., Zhu J., Zhang X., Li Y., Chen J., Liu H. (2023). Progress in traditional Chinese medicine against chronic gastritis: From chronic non-atrophic gastritis to gastric precancerous lesions. Heliyon.

[9] Fu Y., Zhou J.-D., Sang X.-Y., Zhao Q.-T. (2021). Gualou-Xiebai-Banxia decoction protects against type II diabetes with acute myocardial ischemia by attenuating oxidative stress and apoptosis via PI3K/Akt/eNOS signaling. Chin. J. Nat. Med..

[10] Xing Y. (2023). Theory of Treating Coronary Heart Disease by Trichosanthes Peel injection from “Phlegm and Modulating Phlegm-Stasis”. Chin. J. Integr. Tradit. West. Med..

[11] He Y., Dai M.S., Tao L.Y., Gu X., Wang H., Liu P. (2025). Pericarpium Trichosanthis Inhibits TGF-β1-Smad3 Pathway-Induced Cardiac Fibrosis in Heart Failure Rats via Upregulation of microRNA-29b. J. Gene Med..

[12] Chu D., Zhang Z. (2018). Trichosanthis Pericarpium Aqueous Extract Protects H9c2 Cardiomyocytes from Hypoxia/Reoxygenation Injury by Regulating PI3K/Akt/NO Pathway. Molecules.

[13] Zhao L., Zhang H., Li N., Chen J., Xu H., Wang Y., Liang Q. (2023). Network pharmacology, a promising approach to reveal the pharmacology mechanism of Chinese medicine formula. J. Ethnopharmacol..

[14] Yang M., Chen Y., Zhao T., Wang Z. (2020). Effect of astaxanthin on metabolic cataract in rats with type 1 diabetes mellitus. Exp. Mol. Pathol..

[15] Al-Amran F.F., Shahkolahi M. (2014). Oxytocin ameliorates the immediate myocardial injury in heart transplant through down regulation of the neutrophil dependent myocardial apoptosis. Heart Views.

[16] Cao J., Li J., Gu Z., Niu J.-J., An G.-S., Jin Q.-Q., Wang Y.-Y., Huang P., Sun J.-H. (2023). Combined metabolomics and machine learning algorithms to explore metabolic biomarkers for diagnosis of acute myocardial ischemia. Int. J. Leg. Med..

[17] Sun G.-B., Wang M., Xiao J., Sun X.-B. (2011). Protective effects of cynaroside against H_2_O_2_-induced apoptosis in H9c2 cardiomyoblasts. J. Cell. Biochem..

[18] Wang Y., Yan H., Zhao L., He X.-L., Bao T.-R., Sun X.-D., Yang Y.-C., Zhu S.-Y., Gao X.-X., Wang A.-H. (2023). An integrated network pharmacology approach reveals that Darutigenol reduces inflammation and cartilage degradation in a mouse collagen-induced arthritis model by inhibiting the JAK-STAT3 pathway. J. Ethnopharmacol..

[19] Zhu L.-L., Cao G.-Y., Jia L.-Y., Zheng G., Zhang L., Sheng P., Meng Z.-Q., He X., Zhang C.-F., Wang C.-Z. (2022). Muscone suppresses myocardial ischemia damage by regulating PI3K/Akt signaling pathway. Biochim. Biophys. Acta Mol. Basis Dis..

[20] Yuehong Z. (2017). Study on Anti-apoptotic Mechanism of Danshen Yin Extract A through the Pl3K-Akt Signalingpathway. Tradit. Chin. Drug Res. Clin. Pharmacol..

[21] Wu D., Mennerich D., Arndt K., Sugiyama K., Ozaki N., Schwarz K., Wei J., Wu H., Bishopric N.H., Doods H. (2009). Comparison of microsomal prostaglandin E synthase-1 deletion and COX-2 inhibition in acute cardiac ischemia in mice. Prostaglandins Other Lipid Mediat..

[22] Bianchi C., Li J., Simons M., Li J., Sellke F.W., Métais C. (2001). Serotonin-induced human coronary microvascular contraction during acute myocardial ischemia is blocked by COX-2 inhibition. Basic Res. Cardiol..

[23] Faubel S., Edelstein C.L. (2005). Caspases as drug targets in ischemic organ injury. Curr. Drug Targets-Immune Endocr. Metab. Disord..

[24] Liu Z., Li Z., Liu X. (2002). Effect of ginsenoside Re on cardiomyocyte apoptosis and expression of Bcl-2/Bax gene after ischemia and reperfusion in rats. J. Huazhong Univ. Sci. Technol. Med. Sci..

[25] Su M., Zhu L., Zhang Y., Paknejad N., Dey R., Huang J., Lee M.-Y., Williams D., Jordan K.D., Eng E.T. (2020). Structural Basis of the Activation of Heterotrimeric Gs-Protein by Isoproterenol-Bound β1-Adrenergic Receptor. Mol. Cell.

[26] Wang Y., Zhu D., Zhang J., Yu B. (2013). Protective effects of Shengmai lvophilized powder on isoproterenol-induced mvocardial is chemie iniury in mice. Pharmacol. Clin. Chin. Mater. Medica.

[27] Wang K., Wu S., Cui S., Xiang S., Wu X., Zhou M. (2018). Effect of Electroacupuncture on Hippocampal IL-6, IL-1 β, TNF-α and Norepinephrine Levels in Acute Myocardial Ischemia Rats. Zhen Ci Yan Jiu.

[28] Dong Z. (2018). Effect of Huayu Qutan Recipe on the Expression of Myocardial Structure.Electrocardiogram of ST, MDA, SOD, LDH, CK in Blood Serum of AMl Rat. J. Pract. Tradit. Chin. Intern. Med..

[29] Dawson E.A., Shave R., George K., Whyte G., Ball D., Gaze D., Collinson P. (2005). Cardiac drift during prolonged exercise with echocardiographic evidence of reduced diastolic function of the heart. Eur. J. Appl. Physiol..

[30] Henein M.Y., Vancheri S., Longo G., Vancheri F. (2022). The Role of Inflammation in Cardiovascular Disease. Int. J. Mol. Sci..

[31] Dinarello C.A. (2011). A clinical perspective of IL-1β as the gatekeeper of inflammation. Eur. J. Immunol..

[32] Mourouzis K., Oikonomou E., Siasos G., Tsalamadris S., Vogiatzi G., Antonopoulos A., Fountoulakis P., Goliopoulou A., Papaioannou S., Tousoulis D. (2020). Pro-inflammatory Cytokines in Acute Coronary Syndromes. Curr. Pharm. Des..

[33] Hoffmeister H.M., Ehlers R., Büttcher E., Steinmetz A., Kazmaier S., Helber U., Szabo S., Beyer M.E., Seipel L. (2003). Relationship between minor myocardial damage and inflammatory acute-phase reaction in acute coronary syndromes. J. Thromb. Thrombolysis.

[34] Yuan Z., Wang L., Chen J., Su W., Li A., Su G., Liu P., Zhou X. (2021). Electrochemical strategies for the detection of cTnI. Analyst.

[35] Hou D., Fu H., Zheng Y., Lu D., Ma Y., Yin Y., Zhang L., Bao D. (2022). Uncoupling protein 1 knockout aggravates isoproterenol-induced acute myocardial ischemia via AMPK/mTOR/PPARα pathways in rats. Transgenic Res..

[36] Song F., Li H., Sun J., Wang S. (2013). Protective effects of cinnamic acid and cinnamic aldehyde on isoproterenol-induced acute myocardial ischemia in rats. J. Ethnopharmacol..

[37] Iswari R.S., Dafip M., Purwantoyo E. (2021). Malondialdehyde (MDA) Production in Atherosclerosis Supplemented with Steamed Tomato. Pak. J. Biol. Sci..

[38] Bigler M.R., Zimmermann P., Papadis A., Seiler C. (2021). Accuracy of intracoronary ECG parameters for myocardial ischemia detection. J. Electrocardiol..

