# Supplementary material for: Pericarpium Trichosanthis Injection Protects Isoproterenol-Induced Acute Myocardial Ischemia via Suppressing Inflammatory Damage and Apoptosis Pathways
Source: Biomolecules. 2025 Apr 24;15(5):618. doi: 10.3390/biom15050618 (PMC12108571; doi:10.3390/biom15050618)
Supplement: Supplementary file 1 [file biomolecules-15-00618-s001.zip › biomolecules-3564512-supplementary.pdf]

Note：

Caspase-9(46kDa)

Caspase-3(32kDa)

Bcl-2(24kDa)

Bax(21kDa)

GAPDH:38kDa

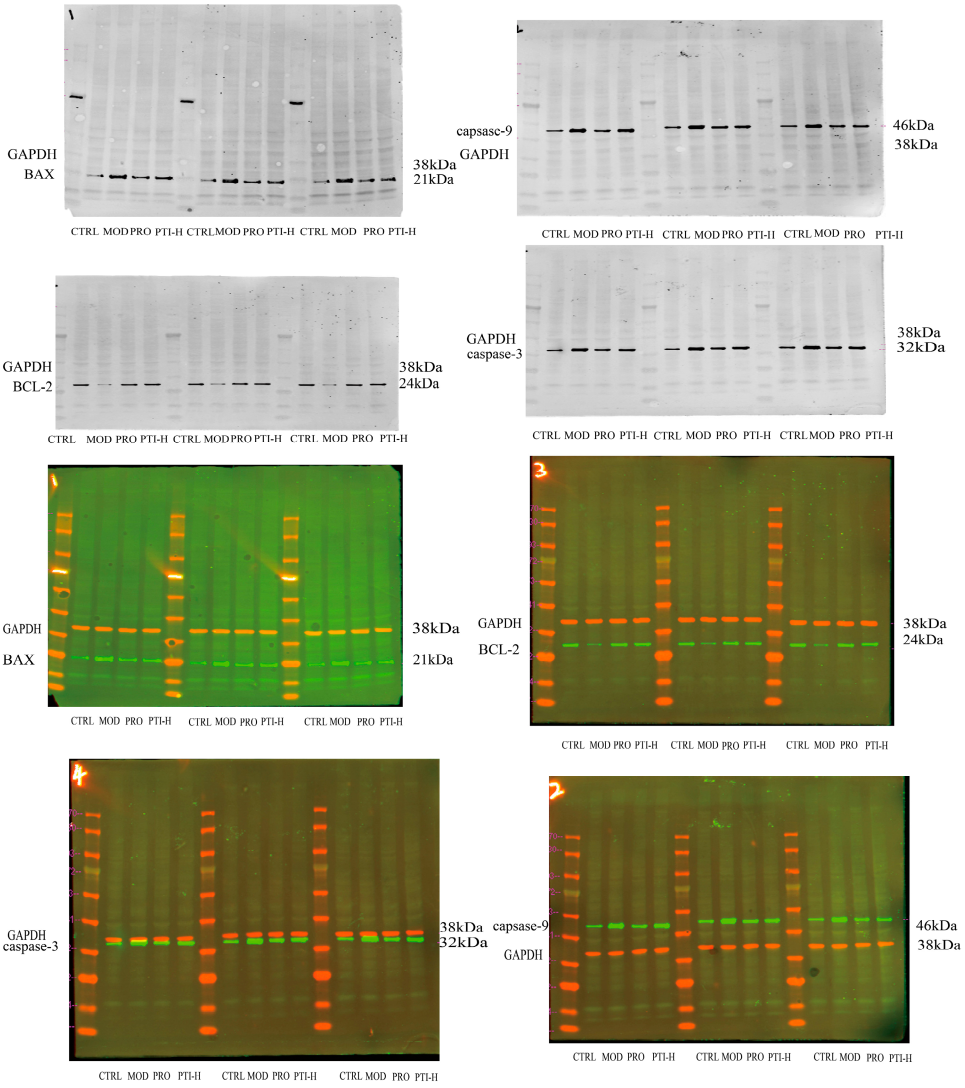

Figure S1: Original Western blot images.

Table S1： The 3 times Molecular docking binding energies results (kcal/mol)）

target proteins / compounds	AKT1	BCL-2	CASPASE-3	EGFR	MAPK3	MMP9	PTGS2	SRC	STAT3
Ciryneol C	-4	-4.5	-4.2	-4.4	-4.2	-4.9	-4.4	-3.8	-4.5
	-4	-3.5	-4.7	-7	-4.9	-5	-5.2	-4.1	-3.9
	-3.1	-5	-3.8	-5.1	-5.3	-5	-4.5	-3.7	-4
Ciryneol C（ average）	-3.7	-4.3	-4.2	-5.5	-4.8	-5.0	-4.7	-3.9	-4.1
Cynanoside M	-5.4	-5.9	-6.2	-8.1	-8.6	-6.6	-9.7	-6.3	-7
	-6.2	-5.9	-8	-7.7	-7.5	-6.2	-7.7	-6.5	-6.6
	-5.5	-6.1	-6.6	-8.6	-8.1	-7.6	-8.5	-6.2	-7.2
Cynanoside M（ average）	-5.7	-6.0	-6.9	-8.1	-8.1	-6.8	-8.6	-6.3	-6.9
Darutigenol	-5.3	-5.5	-6.8	-7.3	-6.2	-6.6	-6.9	-6.1	-6.6
	-5.9	-4.9	-6.4	-8.8	-7.3	-5.6	-7.5	-5.4	-6
	-5	-5.5	-6.4	-7.8	-7.3	-5.9	-8.6	-5.6	-6.3
Darutigenol（ average）	-5.4	-5.3	-6.5	-8.0	-6.9	-6.0	-7.7	-5.7	-6.3
n-Butyl-β-D-fructopyranoside	-4.5	-4.5	-4.5	-5.7	-5.3	-5.4	-5.4	-4.9	-5
	-4.7	-4.5	-4.7	-7.1	-5.1	-5.3	-5.2	-4.6	-5
	-3.4	-4.3	-4.7	-6	-5.1	-5.3	-5.4	-4.8	-4.7
n-Butyl-β-D-fructopyranoside（ average）	-4.2	-4.4	-4.6	-6.3	-5.2	-5.3	-5.3	-4.8	-4.9
Pseudoaspidin	-4.6	-5.1	-5.4	-6.3	-5.8	-5.4	-6.4	-5.3	-5.5
	-4.6	-5.5	-5	-5.8	-5.3	-5.7	-6.1	-4.6	-5
	-4.5	-6	-5.4	-6.6	-5.3	-6.6	-6.1	-5.4	-5.4
Pseudoaspidin（ average）	-4.6	-5.5	-5.3	-6.2	-5.5	-5.9	-6.2	-5.1	-5.3
